# 

**Published:** 1868-05-15

**Authors:** 


*CONTENTS:*

A New Anatomical Feature in Human Blood Corpuscles. By J. W. Freer, M. D. .... 315
Treatment of Diphtheria. By DeLaskie Miller, M. D. .... 316
Calcareous Degeneration of the Pericardium. By Curtis T. Fenn, M. D., Chicago .... 321

FOREIGN CORRESPONDENCE:

Letter from Vienna. By Dr. Samuel Cole .... 323

CORRESPONDENCE:

Letter from Philadelphia. By E. R. H .... 327

BOOK NOTICES:

Atlas of Venereal Diseases. By A. Cullerier .... 329
A Complete List of the Muscles of the Human Body. By William Little, M. D .... 330
A Manual of the Dissection of the Human Body. By Luther Holden, F. R. C. S .... 331
Therapeutics and Materia Medica. By Alfred Stille, M. D. .... 331
Sanitary Institutions during the Austro-Prussian-Italian Conflict. By Thomas W. Evans, M. D. .... 332

SOCIETY REPORTS:

Proceedings of the Chicago Medical Society .... 332

EDITORIAL:

Meeting of the American Medical Association .... 344
Publisher's Notices .... 346

